# Genetic Analysis of Major Production and Reproduction Traits of Korean Duroc, Landrace and Yorkshire Pigs

**DOI:** 10.3390/ani11051321

**Published:** 2021-05-05

**Authors:** Mahboob Alam, Hyuk-Kee Chang, Seung-Soo Lee, Tae-Jeong Choi

**Affiliations:** Animal Breeding and Genetics Division, National Institute of Animal Science, Cheonan-si 31000, Korea; mahboob@korea.kr (M.A.); genemap@korea.kr (S.-S.L.)

**Keywords:** animal model, genetic parameters, production trait, reproduction trait, Korean Duroc pig, Korean Landrace pig, Korean Yorkshire pig

## Abstract

**Simple Summary:**

Korean purebred pigs are generally selected according to their production and reproduction traits in order to optimize commercial pig performance. It is, therefore, crucial to assess the genetic parameters of these traits to understand their genetic potential. For this reason, we analyzed three production traits (average daily gain, ADG; days to 105 kg weight, DAYS105; backfat thickness, BFT) and three reproduction traits (age at first farrowing, AFF; total number of piglets born, TNB; total number born alive, NBA) in Korean Duroc, Landrace and Yorkshire pigs using animal models. We found that production traits were moderately to highly heritable, whereas reproduction traits were mostly lowly heritable. Evidence of selection improvement of DAYS105 and ADG without increasing BFT was also available. Genetically, NBA and TNB were positively and tightly linked, which further pointed to the need for careful breeding plans that consider the negative impact of higher TNB over NBA on piglets’ mortality. Furthermore, AFF needs systematic attention in relation to genetic progress. Overall, most of our estimates suggested further improvement possibilities through selection. We argue that current genetic parameters could greatly assist future breeding and selection decisions for performance improvements in Korean pigs for specific purposes.

**Abstract:**

The study aimed to investigate the genetic parameters of the production and reproduction traits of Korean Duroc, Landrace, and Yorkshire pigs. Three production traits, namely average daily gain (ADG), age at 105 kg body weight (DAYS105) and backfat thickness (BFT), and three reproduction traits, namely age at first farrowing (AFF), total number of piglets born (TNB) and number of piglets born alive (NBA), were analyzed. The reproduction dataset was based on first-parity gilts only. However, the production dataset comprised pigs of both sexes. Genetic parameters were estimated from individual datasets using a multiple-trait animal model in AIREMLF90 software. The heritability values of ADG, DAYS105 and BFT were 0.34–0.36, 0.41–0.44 and 0.38–0.48, respectively, across breeds. Heritability values for AFF, TNB and NBA were 0.07–0.14, 0.09–0.11 and 0.09–0.10, respectively. Strong genetic correlations existed between ADG and DAYS105 (−0.97) and between TNB and NBA (0.90 to 0.96). In line with breeding goals, all productive traits in Duroc pigs, and all reproduction traits except AFF in Landrace and Yorkshire pigs, also showed noticeable improvements in recent years. In conclusion, we believe that our findings on economic traits would greatly assist future pig breeding decisions in Korea.

## 1. Introduction

Both production and reproduction traits are economically significant traits in pig production. In commercial environments, it is preferred that pigs eat less, grow fast, and produce more lean meat [1]. Therefore, pig breeding programs often consider production traits that stimulate animal growth, increase lean meat and decrease production costs [2,3]. In this respect, average daily gain (ADG), age to reach 105 kg body weight (DAYS105), backfat thickness (BFT) and feed efficiency traits are of primary interest. Since feed efficiency traits are difficult to measure, other traits such as ADG, DAYS105 and BFT are used instead as indirect indicator traits [4,5]. These traits have been reported to be strongly positively correlated with feed efficiency [5]. However, for reproduction performance, pig breeders often target traits such as age at first farrowing (AFF), total number of piglets born (TNB) and number of piglets born alive (NBA), which can improve lifetime production traits and, subsequently, lead to more productive, profitable and healthier sows [6,7]. For instance, AFF is shown to have a favorable effect on the lifetime performance of sows [8,9,10]. Similarly, the impacts of TNB and NBA on subsequent parities and the high longevity of sows have been reported [11,12]. In essence, an efficient pig breeding program should consider both categories of traits to ensure the optimal lifetime performance of pigs.

Based on the above viewpoints, Korean pigs are also bred for production and reproduction improvements. Generally, Korean Duroc pigs are selected for higher production performance, whereas Landrace and Yorkshire pigs are chosen for reproduction improvements. According to current practices, Korean pig selection is based mainly on DAYS105, BFT, TNB and NBA. Therefore, many of these traits have been studied previously in Korean pigs [13,14,15]. However, DAYS105 was not assessed extensively due to its recent adoption in the breeding program. Regarding Korean pigs, there is also a lack of in-depth knowledge of ADG and AFF. Nonetheless, the routine importation of pigs into existing pig populations in the last decade has also changed population structures. Given the current status, a review of the genetic potential of the current Korean pig populations is urgently needed.

In this regard, genetic parameters such as heritability and genetic correlations are fundamental, and they are the most valuable tools to evaluate animals’ genetic merit. These parameters indicate the possible genetic worth of traits within animals, which could be exploited through breeding and selection. However, genetic parameters are known to be specific to breed, population or environmental circumstances [16,17]. In other words, these parameters could be changed through the selection and inclusion of superior genetic materials in a population. Therefore, a detailed analysis of these population parameters in Korean pigs would be of great significance.

Therefore, we investigated the enetic parameters of Korean Duroc, Landrace and Yorkshire pigs for major production and reproduction traits using relatively larger datasets. We also assessed the genetic progress of Korean pigs over time. We suggest that a better understanding of their genetic potential will provide us with valuable resources for future pig improvement initiatives.

## 2. Materials and Methods

### 2.1. Animals and Phenotypes

The present study was conducted on Korean Duroc, Landrace and Yorkshire pigs raised on various breeding farms. Data on three production traits (average daily gain, ADG; days at 105 kg, DAYS105; backfat thickness, BFT) and three reproduction traits (age at first farrowing, AFF; total number of piglets born, TNB; number of piglets born alive, NBA) were provided by the Korea Animal Improvement Association, Seoul, Korea, and Korea Pork Producers Association, Seoul, Korea. The production traits dataset consisted of records from 115,501 Duroc, 116,870 Landrace and 368,021 Yorkshire pigs, born between 2000 and 2020, irrespective of sex. All piglets underwent a performance test program to obtain production traits. The performance testing program was started after weaning of piglets while weighing about 30 kg. First, all piglets were grouped by sex. Next, each group was raised separately to reach a target body weight of 105 kg. Performance testing for production traits included at least four (4) pigs per litter.

During animal testing, each pig was weighed on a scale. ADG was easily calculated from the start and end weight of the pigs, considering their linear growth during the test period. Note that we only considered weights between 90 and 120 kg collected during performance testing, as an exact 105 kg weight was difficult to obtain in practice. Then, these weights were readjusted to 105 kg body weight, and an adjusted age at 105 kg (DAYS105) trait was derived. The equation for obtaining DAYS105, provided by the Korean Swine Performance Recording Standards (KSPRS), was as follows:

(1) $$\mathrm{DAYS}105={\mathrm{AGE}}_{t}+\frac{\left(105-{\mathrm{BWT}}_{t}\right)\times \left({\mathrm{AGE}}_{t}-38\right)}{{\mathrm{BWT}}_{t}}$$

where AGE*_t_* is the actual age in days at the *t*th test day, BWT*_t_* is the actual body weight (kg) at the *t*th test day and 38 is the constant term for correction for age.

For BFT, first, three initial measurements from the shoulder (at fourth thoracic vertebrae), mid-back (last thoracic vertebrae) and loin (last lumbar vertebrae) regions were taken using A-mode ultrasound scanners and averaged. Next, the final BFT phenotype was obtained through adjusting the initial average for the target 105 kg body weight using the equation given by KSPRS:

(2) $$\mathrm{BFT}={\mathrm{BFT}}_{t}+\frac{\left(105-{\mathrm{BWT}}_{t}\right)\times {\mathrm{BFT}}_{t}}{{\mathrm{BWT}}_{t}-11.34}$$

where BFT*_t_* is the actual backfat thickness (mm) at the *t*th test day, BWT*_t_* is the actual body weight (kg) at the *t*th test day and 11.34 is the constant term for correction for BWT*_t_* when backfat thickness is 0 cm.

On the other hand, the dataset of the three reproduction traits included first-parity phenotypes from gilts only. Reproduction records of 8906 Duroc, 11,758 Landrace and 40,171 Yorkshire gilts, born between 1992 and 2020, were analyzed. The AFF of all of these gilts ranged between 324 and 438 days. This AFF restriction reflects the assumptions that each female would reach its first heat between 7 and 8 months of age, would conceive within five consecutive estrous cycles (approx. 21 days per cycle) and would farrow after a gestation period of 114 days. We also defined TNB as the total number of piglets born during farrowing, which included all of the normal, stillborn, mummified, deformed and underweight piglets. However, NBA only accounted for live piglets during farrowing and excluded both stillborn and mummified piglets.

### 2.2. Animal Pedigree

The pedigrees related to phenotyped animals were collected from the Korea Animal Improvement Association. The pedigrees for animals with production traits comprised 131,603 animals that were Duroc, 137,538 animals that were Landrace and 415,483 animals that were Yorkshire. Similarly, the pedigrees for reproduction traits comprised 31,603, 37,538 and 15,483 animals that were Duroc, Landrace and Yorkshire pigs, respectively. In all three breeds, the number of generations traced back in the pedigree for production and reproduction datasets was 27 and 26, respectively.

### 2.3. Statistical Analyses

All three pig breeds were analyzed separately for each set of production and reproduction traits. The (co)variance components of traits were estimated through multiple-trait models using the AIREMLF90 software package [18]. The animal models for production traits included a fixed effect of sex (SEX) and a combined fixed effect of the herd, birth year and birth season (HYS), irrespective of breed. Each model also included the random additive genetic effects of animals. The mixed-model equation (MME) for the analysis of production traits in matrix notation was as follows:

(3) y=Xb+Za+e

where y is the vector of observations for multiple production traits, b is the vector of fixed effects related to SEX and HYS, a is the vector related to random direct additive genetic effects of animals and e is the vector related to random residuals. X and Z are matrices relating to observations of the factors in the model. Our assumed (co)variance matrices for random and residual effects were Var (*a*) = $G_{0}⊗A$ and Var (*e*) = $R_{0}⊗I$, where $G_{0}$, A, $R_{0}$ and I are the additive genetic (co)variance matrix between traits, Wright’s numerator relationship matrix indicating additive genetic relationships among individuals, residual (co)variance matrix between traits and identity matrix, respectively.

On the other hand, the animal models for reproduction traits included a similar fixed effect of HYS and a fixed effect of farrowing year-season, irrespective of breeds. Each model also included the random additive genetic effects of dams. The MME for the multiple-trait animal model was the same as given above for production traits.

Total phenotypic variance ($\sigma_{p}^{2}$) is calculated as $\sigma_{p}^{2}=\sigma_{a}^{2}+\sigma_{e}^{2}$, where the $\sigma_{a}^{2}$ and $\sigma_{e}^{2}$ parameters are the additive genetic variance and the random residual variance, respectively. Trait heritability (h^2^) was computed by the equation, h^2^ = $\sigma_{a}^{2}$/$\sigma_{p}^{2}$. The genetic correlation (r_g_) between two traits was estimated as

(4) $$r_{g}=\frac{\sigma_{a_{1}a_{2}}}{\sqrt{\sigma_{a_{1}}^{2}\times \sigma_{a_{2}}^{2}}}$$

where the $\sigma_{a_{1}}^{2}$ and $\sigma_{a_{2}}^{2}$ parameters are genetic variance estimates of traits 1 and 2, respectively, and $\sigma_{a_{1}a_{2}}\;$ is the genetic covariance between two traits. Similarly, we also estimated the phenotypic correlation (r_p_) between traits using phenotypic variances and covariances of two traits. Note that phenotypic correlation was provided for completeness purposes only. We further obtained the approximated standard error (SE) of genetic parameter estimates (h^2^ and r_g_) from (co)variance components using the AIREMLF90 option (se_covar_function), which uses a Monte Carlo method for the computation of SE, as proposed by Houle and Meyer [19]. The ratio of a trait’s genetic standard deviation ($\sigma_{a}$) and its mean were expressed as the additive genetic coefficient of variation (CV_g_), according to Houle [20]. We also obtained the genetic trend in traits using the averaged estimated breeding value (EBV) of animals based on their birth year. The distribution of EBVs for production and reproduction traits is given in Figure S1 and Figure S2.

## 3. Results

### 3.1. Descriptive Statistics

Table 1 presents a descriptive summary of the ADG, DAYS105 and BFT traits of Korean Duroc, Landrace and Yorkshire pigs. Duroc pigs showed an average ADG of 661.11 ± 60.42 g, the highest among all breeds in our study. The averages of ADG in Landrace and Yorkshire pigs were 643.07 ± 56.82 g and 641.37 ± 57.35 g, respectively. The coefficient of variation (CV) in ADG was slightly higher in Duroc pigs than in other breeds. For DAYS105, Duroc pigs also performed the best among three breeds, showing the lowest duration of 156.31 ± 12.79 days. The other two breeds had slightly higher and similar DAYS105 averages. However, the DAYS105-CVs were somewhat similar across breeds. For BFT, we obtained the lowest average in Duroc pigs (12.55 ± 2.37 mm) and the highest in Yorkshire pigs (13.27 ± 2.53 mm). The BFT-CVs also ranged between 18.89% and 19.75% across breeds.

Similarly, Table 2 presents the descriptive statistics of AFF, TNB and NBA for Korean Duroc, Landrace and Yorkshire pigs. Landrace pigs showed the lowest average AFF value of 362.73 ± 23.08 days. However, Duroc females were the most late-farrowing animals. Despite some differences in average AFF, its CV values were smaller and similar across breeds. For TNB, the average of 12.07 ± 3.40 piglets in Yorkshire was the highest. The lowest TNB average was in Duroc pigs. For the same trait, Landrace showed an intermediate mean to the other two breeds. Variations in TNB ranged between 27.60% and 28.43%. As for TNB, the mean NBA of 11.04 ± 3.18 piglets was the highest in Yorkshire pigs, whereas NBA in Durocs was 8.28 ± 2.55 piglets, also being the lowest. The NBA-CV was 30.77% in Duroc pigs, while that of Yorkshire pigs was nearly 3% less variable.

### 3.2. Variance Components and Genetic Parameter Estimates

Table 3 shows the variance components (genetic and residual), heritability (h^2^) and genetic correlation (r_g_) estimates of ADG, DAYS105 and BFT traits. Our results showed that the h^2^ of ADG was 0.36 ± 0.01 in Duroc, 0.36 ± 0.01 in Landrace and 0.34 ± 0.00 in Yorkshire pigs. The genetic variation in ADG was about 4% in all pigs. For DAYS105, the h^2^ values were 0.44 ± 0.01, 0.42 ± 0.01 and 0.41 ± 0.00 in Duroc, Landrace and Yorkshire pigs, respectively. Their DAYS105-CV_g_ estimates were also low and similar (0.04). We also found that the h^2^ of BFT was 0.38 ± 0.01 in Duroc, 0.36 ± 0.01 in Landrace and 0.45 ± 0.00 in Yorkshire. The genetic variation range in BFT was 10–12% across the breeds, also being the highest among production traits. Our h^2^ estimates indicated that all three traits were moderately to highly heritable across Korean breeds. Our estimates also indicated that h^2^ values in Landrace were either equal to or higher than those of the other two breeds. We also observed a similar and strongly negative r_g_ estimate between ADG and DAYS105 (r_g_: −0.97) in all pigs in this study, which indicated their inverse relationship. On the other hand, the association of ADG with BFT was mostly not significantly different from zero. Similarly, DAYS105 and BFT showed very lowly negative r_g_ values, ranging between −0.01 and −0.06 across breeds. These results also indicated that there were almost no genetic associations between DAYS105 and BFT.

In addition, Table 4 shows the variance components (genetic and residual), h^2^ and r_g_ estimates of AFF, TNB and NBA traits in Korean pigs. In this study, the h^2^ value for the AFF trait was 0.14 ± 0.02 in Duroc, 0.07 ± 0.02 in Landrace and 0.12 ± 0.01 in Yorkshire pigs. Genetic variation in AFF was relatively low in all Korean pigs, i.e., CV_g_: 1–2%. For TNB, our h^2^ values in all pigs were low, ranging between 0.09 and 0.11. Similar to TNB, the h^2^ of NBA was low in all three breeds. The AFF-CV_g_ values mostly ranged between 8% and 9% across breeds. For AFF and TNB, both Duroc and Yorkshire showed low, negative r_g_ values, such as −0.01 to −0.02, which were also not significantly different from zero. Landrace pigs, in this regard, exhibited a low and negative but slightly stronger association: −0.23 ± 0.14. Landrace pigs also displayed a somewhat similar, negative r_g_ between AFF and NBA (−0.21 ± 0.14), in which the other two breeds showed no significant association. On the other hand, the association between TNB and NBA was positive and strong in all pigs, i.e., 0.90 to 0.96. This indicated that both traits would have similar genetic backgrounds and were likely to influence each other under selection.

### 3.3. Genetic Trends of Production and Reproduction Traits

**Figure 1:**
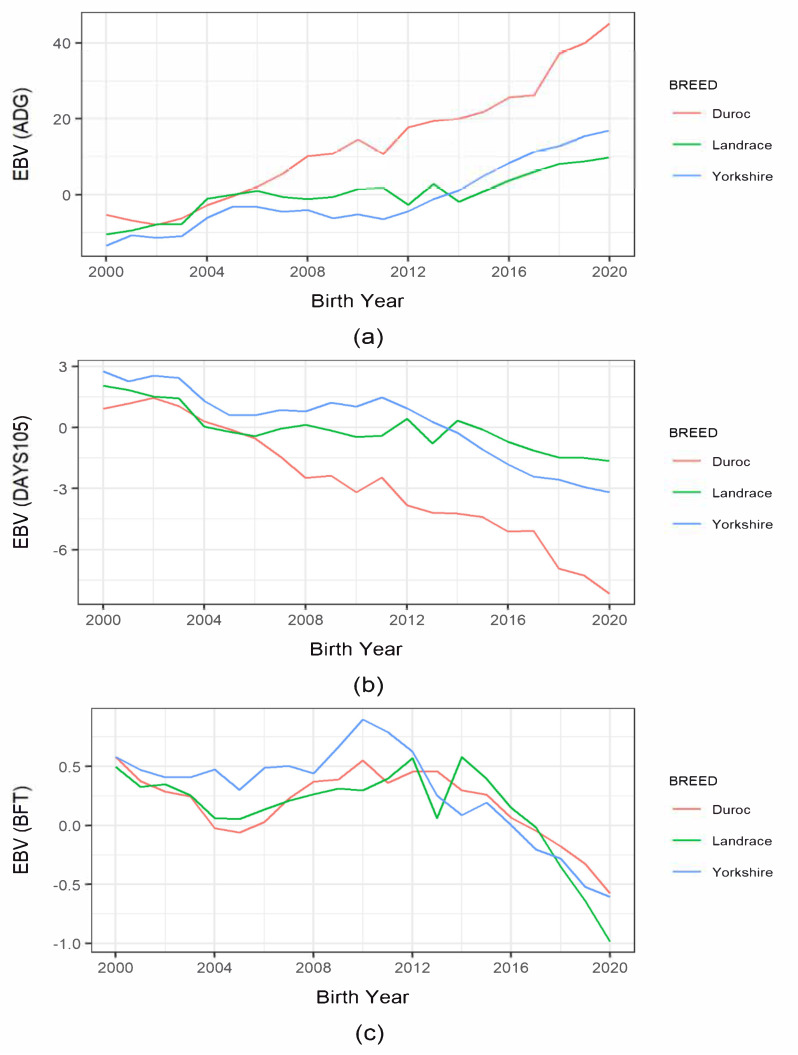
Trend of estimated breeding value (EBV) of production traits in Korean pig breeds by year of births: (**a**) average daily gain (ADG); (**b**) days to 105 kg body weight; (**c**) backfat thickness.

Figure 1 presents the estimated breeding value (EBV) in each pig breed for production traits and subjectively compares their trends. Since 2000, ADG-EBV had increased from −5.23 g to 45.16 g in Duroc pigs. Its EBV almost doubled within recent years. There was also a consistent increase in Landrace and Yorkshire pigs, especially since 2014. Despite the differences in the changes in ADG, all three breeds showed overall genetic improvements. For DAYS105, we found a consistent and noticeable decline in Duroc pigs starting in 2000, i.e., from 0.91 to −8.16 d. Moreover, Landrace and Yorkshire pigs showed a lesser decline in EBVs when compared to Duroc pigs. The BFT-EBV trends were somewhat inconsistent in all breeds until 2014. However, there was an overall consistent and decreasing trend in BFT afterward. Note that, among the three breeds, a noticeable decrease was also found in Landrace pigs, i.e., from 0.57 to −0.99, from 2014.

**Figure 2:**
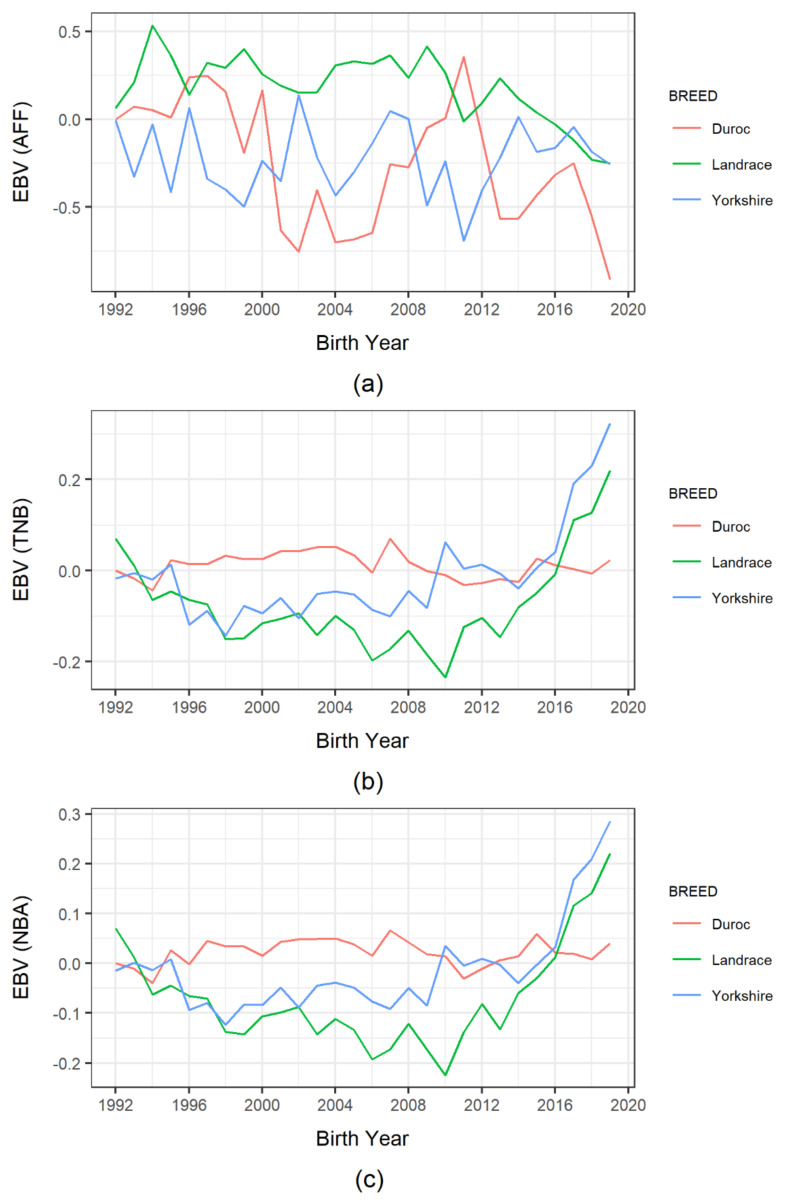
Trend of estimated breeding value (EBV) of reproduction traits in Korean pig breeds by birth year: (**a**) age at first farrowing (AFF); (**b**) total number of piglets born (TNB); (**c**) number of piglets born alive (NBA).

Similarly, Figure 2 shows the trends in animal EBVs for reproduction traits and their subjective comparisons. The genetic response to AFF-EBVs was mainly inconsistent in all breeds, with no apparent visual trends. Similarly, we identified no distinct trends for TNB-EBVs in Landrace and Yorkshire pigs prior to 2014, after which they displayed noticeable upward trends. Moreover, Yorkshire TNB-EBVs were slightly higher than Landrace TNB-EBVs after 2002. However, Duroc pigs exhibited minimal changes in TNB-EBVs over the years. The NBA-EBVs of Duroc pigs also demonstrated no noticeable visual trends. With the other two breeds, their NBA-EBV trends were similar to their TNB-EBV trends, even though Yorkshire pigs mostly performed better than Landrace pigs.

## 4. Discussion

### 4.1. Heritability of Production Traits

The current study showed that the heritability of ADG was somewhat moderate across Korean breeds, i.e., h^2^: 0.32–0.34. The study conducted by Clutter [21], which summarized multiple reports, showed that heritability could vary widely for ADG across studies (i.e., 0.03 to 0.49). Even though that study reported a wide h^2^ range for ADG, our values showed substantial agreement with those values. We also observed further general agreement with other reports. Notably, some studies reporting h^2^ of 0.35 ± 0.01 in Korean Duroc pigs, 0.397 in Danish Duroc and 0.38 ± 0.17 in Thai Landrace pigs further verified our results [15,22,23]. On the other hand, we found other works reporting slightly higher h^2^ values in similar breeds [1,14,24,25,26]. Similarly, some studies were also available that presented slightly lower h^2^ values, such as 0.28 in US Duroc pigs and 0.30 in crossbred pigs [27,28]. It was evident that the differences that existed between reports on similar Korean pigs [14,15] could be explained by various factors, such as the differences in data structures (multi-parity records), adopted models and sampling errors [29].

Our findings for DAYS105 revealed moderate heritability values across Korean breeds, ranging from 0.39 to 0.44. We found a lot of earlier reports in Korean pigs based on days to 90 kg (DAYS90), but none for DAYS105. To our knowledge, this is the first study to evaluate DAYS105 in Korean pigs. Due to the lack of comparison among Korean pigs, we compared our findings to those based on DAYS90 instead. In this regard, we observed that Choi et al. reported similar h^2^ values for DAYS90 (0.36 to 0.40) using similar pigs [30]. Another work considering DAYS90 in Korean pigs found more similar values, such as 0.36–0.37 in Duroc, 0.37–0.38 in Landrace and 0.41–0.42 in Yorkshire pigs [13]. Other comparable h^2^ values of DAYS90 include an h^2^ of 0.37 in Duroc and 0.37 to 0.42 in Landrace pigs [15,31]. Furthermore, Hoque et al., who studied DAYS105 in Japanese Duroc pigs, also showed some agreements with our findings [5]. Another variant of the trait (age at 100 kg), in a composite Chinese dam line (Tai Zumu line), had an h^2^ of 0.42 ± 0.02, which is in agreement with our findings [32]. However, a report on DAYS90 revealed some discrepancies, such as high heritability (i.e., 0.46 to 0.56) in Korean Landrace and Yorkshire pigs [14].

In Korean pigs, we also found a moderate h^2^ of BFT (0.38 to 0.48). Numerous studies, on the other hand, yielded h^2^ values that seemed to have a broader range. According to Clutter [21], several studies reported an average h^2^ of 0.49, which was in line with our findings. Lopez et al. also had an h^2^ of 0.35, further supporting our values [15]. In other studies, consistent h^2^ estimates were published in Duroc pigs (0.53 ± 0.15) [33], as well as in Landrace (0.54 ± 0.01) and Yorkshire pigs (0.45 ± 0.01) [14]. Other reports in different breeds showed overall agreement, presenting values between 0.38 and 0.61 [1,27,28,32,34,35,36,37].

### 4.2. Genetic Correlations among Production Traits

In this study, the genetic correlation (r_g_) between production traits was of a similar magnitude across breeds. Our study suggested that ADG and DAYS105 were firmly and oppositely (−0.96 to −0.97) genetically linked. In Duroc pigs, a similar high, opposite correlation (−0.98) was reported previously [15]. Such high r_g_ values essentially indicate that selection for either ADG or DAYS105 could result in favorable changes in both of these traits. We demonstrated a lack of correlation between ADG and BFT across Korean breeds. However, according to a review report, their summarized genetic association values ranged from −0.26 to 0.55 [21]. They also reported an average r_g_ of 0.14, which was closer to our estimates. In the same way, our results reflected those of Lopez et al. [14,15] where they found similar lower correlations in Korean Duroc (−0.05), Landrace (0.00 ± 0.022) and Yorkshire pigs (−0.02 ± 0.014). There was also partial agreement with Miar et al. [28], with their similar lower estimate between ADG and subcutaneous backfat depth in commercial crossbred pigs. Nonetheless, several other published figures, such as those ranging from −0.21 to −0.47 in Akanno et al. [38], −0.19 in Chang et al. [39] and 0.35 ± 0.18 in Ito et al. [33], were found to be inconsistent with ours. In this regard, Clutter [21] stated that the genetic association of these two traits is determined by how closely each of these traits is linked to feed intake versus the ability to partition energy intake towards lean tissue development. Previously, both faster-growing pigs and fatter pigs were linked to higher feed requirements [26], indicating selection challenges for a higher ADG without a higher BFT. In Korean pigs, a near absence of correlation between ADG and BFT may assist in partitioning more energy for body growth than fatness. Furthermore, our estimates of almost no genetic association between DAYS105 and BFT matched those of Choy et al. [13], who reported a range of estimates between DAYS90 and BFT (r_g_: −0.11 and 0.08). In Korean pigs, there were further similarities to those of Lopez et al. [14,15]. However, as noted previously, there are difficulties in comparing DAYS105 results across Korean studies. Many of these studies, on the other hand, corroborated our findings. Such resemblances may point to underlying genetic similarities between two traits. As a result, using DAYS105 instead of DAYS90 in pig evaluations may not significantly impact BFT in Korean pigs. At the same time, the lack of correlation between BFT and other production traits also indicated the need for a separate BFT improvement strategy for pigs.

### 4.3. Heritability of Reproduction Traits

AFF was found to be lowly heritable (0.07 to 0.14) across Korean breeds in our analysis. An Australian study of commercial Large White pigs reported an h^2^ value of 0.10, showing greater agreement [40]. Our h^2^ values were also within the range of 0.04–0.16 values reported in crossbred Landrace × Large White [41] and Finnish Landrace pigs [42]. Other reports, on the other hand, showed little agreement. For example, in Bísaro pigs, the h^2^ of AFF was 0.35 [43]. With a higher h^2^ of 0.44 in sows, Cavalcante-Neto et al. [44] presented further arguments. The h^2^ value of 0.23 found by Akanno et al. [38], based on a meta-analysis study, also reported some disagreement. Besides breed and model differences across studies, other factors such as age ranges for AFF analysis could be partially responsible for such disagreements [38]. Notably, the AFF was inconsistent across studies, e.g., 240–540 or 290–500 days [41,43], whereas it was only 324–438 days in this study. This inconsistency in AFF indicates the disparities in the overall spreads and CVs of the AFF phenotype among studies. Thus, the AFF-h^2^ variability was not unlikely among reports. The low h^2^ of AFF could also be due to the lower genetic variability in Korean pigs. However, there were no existing studies focusing on Korean pigs with which to compare our results regarding the AFF trait. We therefore strongly recommend future analysis of the AFF trait to verify the present estimates through the use of different models and larger datasets. We suggest that necessary precautions be taken while considering the present AFF estimate for breeding decisions. We also found low heritability of TNB in Korean breeds. Numerous published reports displayed general agreement in this regard. Using a single-trait repeatability model, Ogawa et al. [45] obtained an h^2^ of 0.12 for TNB in Landrace and Large White pigs, implying consensus. The results of a genotype marker-based study on Korean Yorkshire pigs also matched our results [46]. The lower h^2^ values in other pigs (e.g., <0.10) further supported our results [47,48,49]. Canadian Yorkshire and Landrace pigs were also not significantly different [50]. However, some inconsistencies existed in the literature, according to a few studies [32,51]. The NBA, like TNB, also had low heritability in this study. In this respect, Ogawa et al. [45] corroborated our study by reporting an h^2^ of 0.12 in Landrace and 0.10 in Large White pigs. Multiple pig studies [46,47,48] also yielded consistent results. Ye et al. [49], using multi-parity records in Large White pigs, also showed a lower NBA h^2^ (0.06) in their pigs. Other reports on Landrace and other pigs suggest further agreement [52,53,54,55]. Nonetheless, a few reports have also registered some variations in NBA h^2^ [32,51]. Despite some inconsistencies among breeds, most evidence suggests that reproduction traits, especially TNB and NBA, are essentially lowly heritable traits. At the same time, Korean pig populations are not significantly dissimilar to others. To summarize, a faster genetic improvement in any of the reproduction traits is not expected in Korean pigs due to their lower heritability values. Therefore, Korean pig breeding strategies should focus on increasing animals’ genetic variabilities to achieve desirable selection responses, especially for the AFF trait.

### 4.4. Genetic Correlations among Reproduction Traits

In Korean pig breeds, the genetic association estimates of AFF with both TNB and NBA were lowly negative. However, most of these estimates were not significantly different from zero, except for Landrace pigs. This suggests that selection of Landrace pigs against AFF could increase TNB or NBA to some extent, if not much. Moreover, the non-significant correlation of AFF with TNB or NBA in Duroc and Yorkshire pigs suggest no selection improvements in these breeds. The correlation between TNB and NBA was 0.90 or higher in our work. Other studies previously found similar genetic associations [45,49,50,56]. In some reports, correlations in different breeds were almost close to 1, suggesting agreement with ours [32,48]. For this reason, both TNB and NBA could be assumed to have almost similar genetic bases, and an improvement in TNB could have subsequent desired effects on NBA. However, these strong associations should be interpreted carefully, as an increase in TNB could lower piglets’ birth weight and increase post-natal piglet mortality, finally resulting in a decrease in sows lifetime productivity [51]. A lower birth weight could also increase production costs due to additional care management. A better means of counter piglet mortality could be accounting for birth weight while selecting for TNB and NBA. However, as birth weights are not recorded for Korean piglets, we recommend future initiatives regarding the recording of birth weight.

### 4.5. Genetic Trends in Production and Reproduction Traits

Korean Duroc pigs are typically selected for DAYS105 and BFT traits. At the same time, Landrace and Yorkshire pigs are selected for reproduction traits only. Our trend analysis showed somewhat consistent improvements in the breeds for which they were selected. A reason for these improvements could be the positive effect of evaluating animals under the National Pig Network, which has been taking place since 2008. This network essentially merged many sources of pigs into one large genetic pool. In Duroc pigs, we found a positive increase in ADG EBVs, which was likely, even though ADG was not used as a selection tool in this case. This explains the indirect improvement in ADG caused by the selection for DAYS105, which occurs because both traits are strongly and negatively correlated to each other. The reduction in BFT EBVs in Duroc pigs after 2008 also demonstrates the benefit of combined pig evaluation. The notable improvement in Duroc pigs for production traits, caused by direct selection, is readily apparent. On the other hand, the improvements in Landrace and Yorkshire production traits, especially for BFT, remain somewhat unclear, as these traits were not used for animal selection. One assumption may be linked to farmers’ unintentional bias toward larger animals as parents beyond their reproduction traits. As the relationships between production and reproduction traits were not investigated in this study, we strongly recommend future studies to do so in this manner, so that the genetic nature of such improvements is clearly understood. For AFF, we observed no noticeable trend in EBVs in Landrace and Yorkshire pigs. No direct selection for AFF could explain, even partially, this absence of a clear trend. Another reason could be the genetic parameters of AFF, such as the low heritability, meaning greater environmental influences, and the lack of genetic correlations with TNB or NBA. Therefore, animals should be selected directly for AFF improvement. Since 2014, we have also observed consistent improvements in TNB and NBA, especially within Landrace and Yorkshire pigs, as they are directly targeted for reproduction improvement. On the other hand, the lack of consideration of reproduction traits in Duroc pigs, as per their breeding goals, could easily explain their lack of improvement. Irrespective of the type of trait, however, we observed accelerated improvement in pigs over recent years, where the importation of superior animals might have made a significant contribution. According to recent statistics [57], the average annual import between 2007 and 2010 was 1412 pigs, which increased by more than 4-fold between 2011 and 2012. Between 2011 and 2020, the average annual import was also reported as more than 2-fold greater than in previous years. Therefore, we assume that a substantial change in Korean pigs’ performances in the following years was not unlikely.

## 5. Conclusions

In conclusion, our findings revealed that the heritability of productive traits (ADG, DAYS105 and BFT) was moderate to high (0.40–0.52) in Korean pigs. Heritability estimates for reproduction traits (AFF, TNB and NBA) were low. Genetic correlations between productive traits showed that a reduction in DAYS105 could also increase ADG without affecting BFT across breeds, especially in Duroc pigs. Similarly, as TNB and NBA had a strong and positive relationship, an increase in NBA could also increase TNB. A higher TNB, on the other hand, may present additional problems in the form of increased piglet mortality and reduced sow productivity. As a result, careful selection for higher TNB and NBA phenotypes is required to optimize the NBA. TNB or NBA, on the other hand, had no impact on AFF. There was also no noticeable improvement in AFF, highlighting the lack of animal selection for the trait. The present study suggests that careful breeding decisions are required to formulate breeding goals based on the genetic associations between traits. We posit that our study on DAYS105 could foster a better understanding of Korean pigs’ production traits. The present findings for all other traits may also have an impact on future breeding decisions.

## Figures and Tables

**Table 1:** Descriptive statistics ^1^ for production traits in Korean Duroc, Landrace and Yorkshire pigs.

Breed	Trait	N	Mean	SD	Minimum Value	Maximum Value	CV
Duroc	ADG	115,501	666.11	60.42	470.00	906.80	9.07
	DAYS105	115,501	156.31	12.79	115.42	214.17	8.18
	BFT	115,501	12.55	2.37	1.83	24.10	18.89
Landrace	ADG	116,870	643.07	56.82	468.00	908.20	8.84
	DAYS105	116,870	161.21	12.98	113.60	215.33	8.05
	BFT	116,870	12.68	2.50	1.88	24.18	19.75
Yorkshire	ADG	368,021	641.37	57.35	468.00	908.20	8.94
	DAYS105	368,021	161.36	13.10	115.78	215.33	8.12
	BFT	368,021	13.27	2.53	1.83	24.20	19.07

^1^ ADG, average daily gain (g); DAYS105, days to 105 kg body weight; BFT, backfat thickness (mm); N, total sample size; SD, standard deviation; CV, coefficient of variation.

**Table 2:** Descriptive statistics ^1^ for reproduction traits in Korean Duroc, Landrace and Yorkshire pigs.

Breed	Trait	N	Mean	SD	Minimum Value	Maximum Value	CV
Duroc	AFF	8906	370.86	23.66	324	438	6.38
	TNB	8906	9.28	2.64	1	20	28.43
	NBA	8906	8.28	2.55	1	19	30.77
Landrace	AFF	11,758	362.73	23.08	324	438	6.36
	TNB	11,758	11.53	3.18	1	20	27.60
	NBA	11,757	10.63	2.98	0	20	28.02
Yorkshire	AFF	40,171	368.82	22.95	324	438	6.22
	TNB	40,171	12.07	3.40	1	20	28.13
	NBA	40,167	11.04	3.18	0	20	28.85

^1^ AFF, age at first farrowing (day); TNB, total number of piglets born; NBA, number of piglets born alive; N, total sample size; SD, standard deviation; CV, coefficient of variation.

**Table 3:** Estimates ^1^ of heritability (diagonals) and genetic correlations (upper diagonal) and phenotypic correlation (lower diagonal) of production traits with standard error in Duroc, Landrace and Yorkshire pigs using a multiple-trait animal model.

Breed	Trait	Genetic Parameter Estimates			Genetic Variance	Residual Variance	CV_g_
		ADG	DAYS105	BFT			
Duroc	ADG	0.36 ± 0.01	−0.97 ± 0.00	−0.01 ± 0.02	868.90	1578.00	0.04
	DAYS105	−0.97 ± 0.00	0.44 ± 0.01	−0.01 ± 0.02	41.80	53.54	0.04
	BFT	−0.10 ± 0.00	0.07 ± 0.00	0.38 ± 0.01	1.44	2.31	0.10
Landrace	ADG	0.36 ± 0.01	−0.97 ± 0.00	0.00 ± 0.02	776.60	1386.00	0.04
	DAYS105	−0.97 ± 0.00	0.42 ± 0.01	−0.01 ± 0.02	38.78	54.55	0.04
	BFT	−0.05 ± 0.00	0.04 ± 0.00	0.48 ± 0.01	2.39	2.59	0.12
Yorkshire	ADG	0.34 ± 0.00	−0.97 ± 0.00	0.04 ± 0.01	720.90	1382.00	0.04
	DAYS105	−0.97 ± 0.00	0.41 ± 0.00	−0.06 ± 0.01	38.30	54.81	0.04
	BFT	−0.03 ± 0.00	0.01 ± 0.00	0.45 ± 0.00	2.26	2.74	0.11

^1^ ADG, average daily gain; DAYS105, days to 105 kg body weight; BFT, backfat thickness; CV_g_, genetic coefficient of variation.

**Table 4:** Estimates ^1^ of heritability (diagonals) and genetic correlations (upper diagonal), phenotypic correlation (lower diagonal) of reproduction traits with standard error in Duroc, Landrace and Yorkshire pigs.

Breed	Trait	Genetic Parameter Estimates			Genetic Variance	Residual Variance	CV_g_
		AFF	TNB	NBA			
Duroc	AFF	0.14 ± 0.02	−0.02 ± 0.15	−0.13 ± 0.15	39.96	248.00	0.02
	TNB	0.02 ± 0.01	0.09 ± 0.02	0.90 ± 0.04	0.56	5.83	0.08
	NBA	0.00 ± 0.01	0.86 ± 0.00	0.09 ± 0.02	0.52	5.42	0.09
Landrace	AFF	0.07 ± 0.02	−0.21 ± 0.14	−0.23 ± 0.14	21.08	261.40	0.01
	TNB	−0.01 ± 0.01	0.10 ± 0.02	0.94 ± 0.02	0.87	7.83	0.08
	NBA	−0.03 ± 0.01	0.90 ± 0.00	0.10 ± 0.02	0.77	7.08	0.08
Yorkshire	AFF	0.12 ± 0.01	−0.01 ± 0.06	−0.04 ± 0.06	37.88	277.00	0.02
	TNB	0.00 ± 0.01	0.11 ± 0.01	0.96 ± 0.01	0.99	8.35	0.08
	NBA	0.00 ± 0.01	0.90 ± 0.00	0.09 ± 0.01	0.77	7.75	0.08

^1^ AFF, age at first farrowing; TNB, total number of piglets born; NBA, number of piglets born alive; CV_g_, genetic coefficient of variation.

## Data Availability

Data sharing not applicable.

## Supplementary Materials

The following are available online at https://www.mdpi.com/article/10.3390/ani11051321/s1, Figure S1: Distribution of estimated breeding value for ADG (average daily gain), DAYS105 (days to 105 kg body weight), and BFT (backfat thickness) in three Korean pig breeds: (a–c) Duroc pigs (d–f) Landrace pigs, (g–i) Yorkshire pigs; Figure S2: Distribution of estimated breeding value for AFF (age at first farrowing), TNB (total number of piglets born), and NBA (total number of piglets born alive) in three Korean pig breeds: (a–c) Duroc pigs, (d–f) Landrace pigs, (g–i) Yorkshire pigs.
