# Supplementary material for: Genetic Analysis of Major Production and Reproduction Traits of Korean Duroc, Landrace and Yorkshire Pigs
Source: Animals (Basel). 2021 May 5;11(5):1321. doi: 10.3390/ani11051321 (PMC8147943; doi:10.3390/ani11051321)
Supplement: Supplementary file 1 [file animals-11-01321-s001.zip › animals-1203984 supplementary.pdf]

animals

MDPI

Article

Genetic Analysis of Major Production and Reproduction Traits of Korean Duroc, Landrace and Yorkshire Pigs

Mahboob Alam †, Hyuk-Kee Chang *,†, Seung-Soo Lee and Tae-Jeong Choi *

Animal Breeding and Genetics Division, National Institute of Animal Science, Cheonan-si 31000, Korea; mahboob@korea.kr (M.A.); genemap@korea.kr (S.-S.L.)

* Correspondence: huk0505@korea.kr (H.-K.C.); choi6695@korea.kr (T.-J.C.); Tel.: +82-580-3353 (H.-K.C.); +82-580-3362 (T.-J.C.)

† These authors contributed equally to this work.

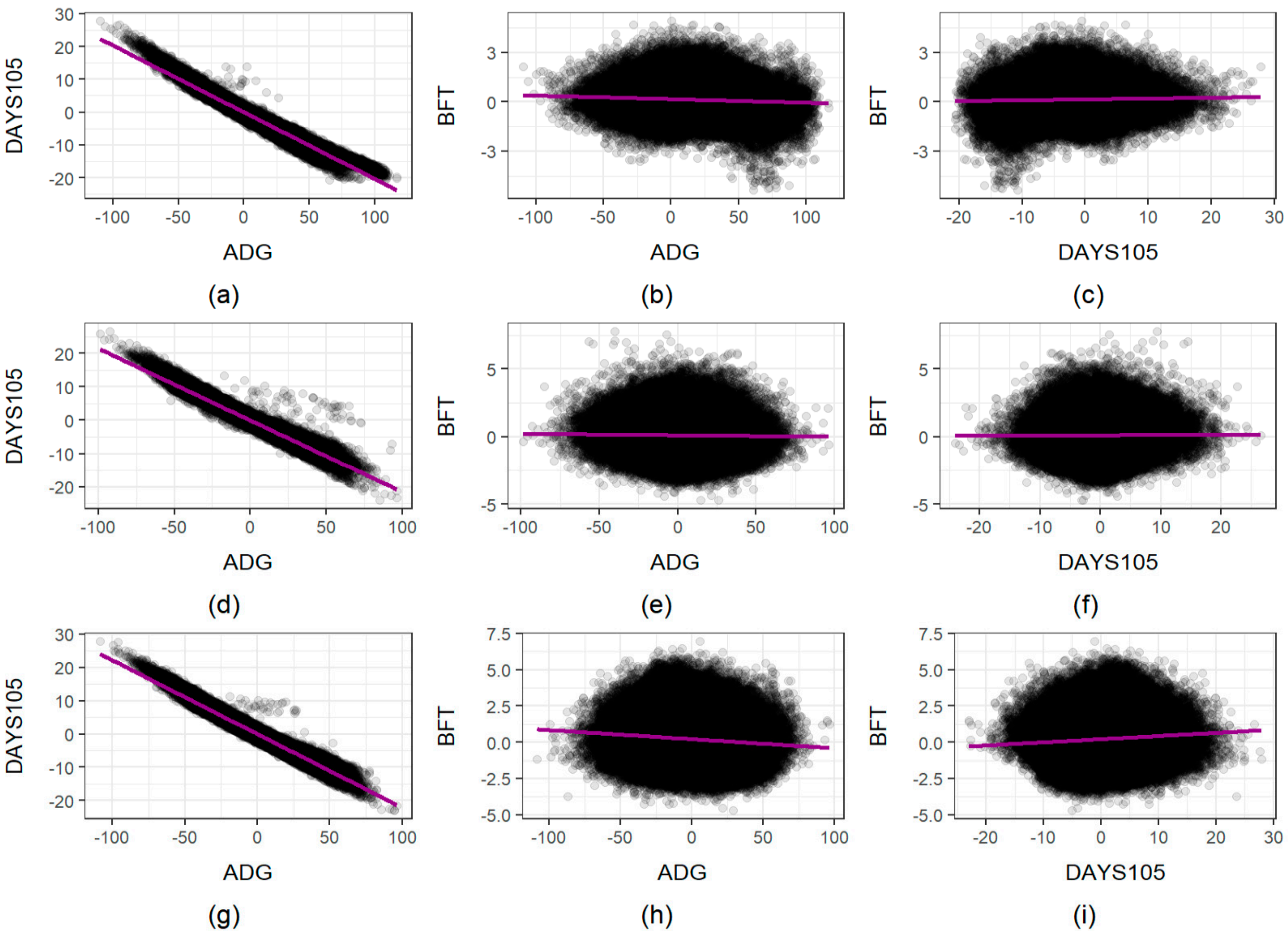

Figure S1. Distribution of EBV for ADG (average daily gain), DAYS105 (days to 105 kg body weight), and BFT (backfat thickness) in three Korean pig breeds. (**a–c**) Duroc pigs, (**d–f**) Landrace pigs, (**g–i**) Yorkshire pigs.

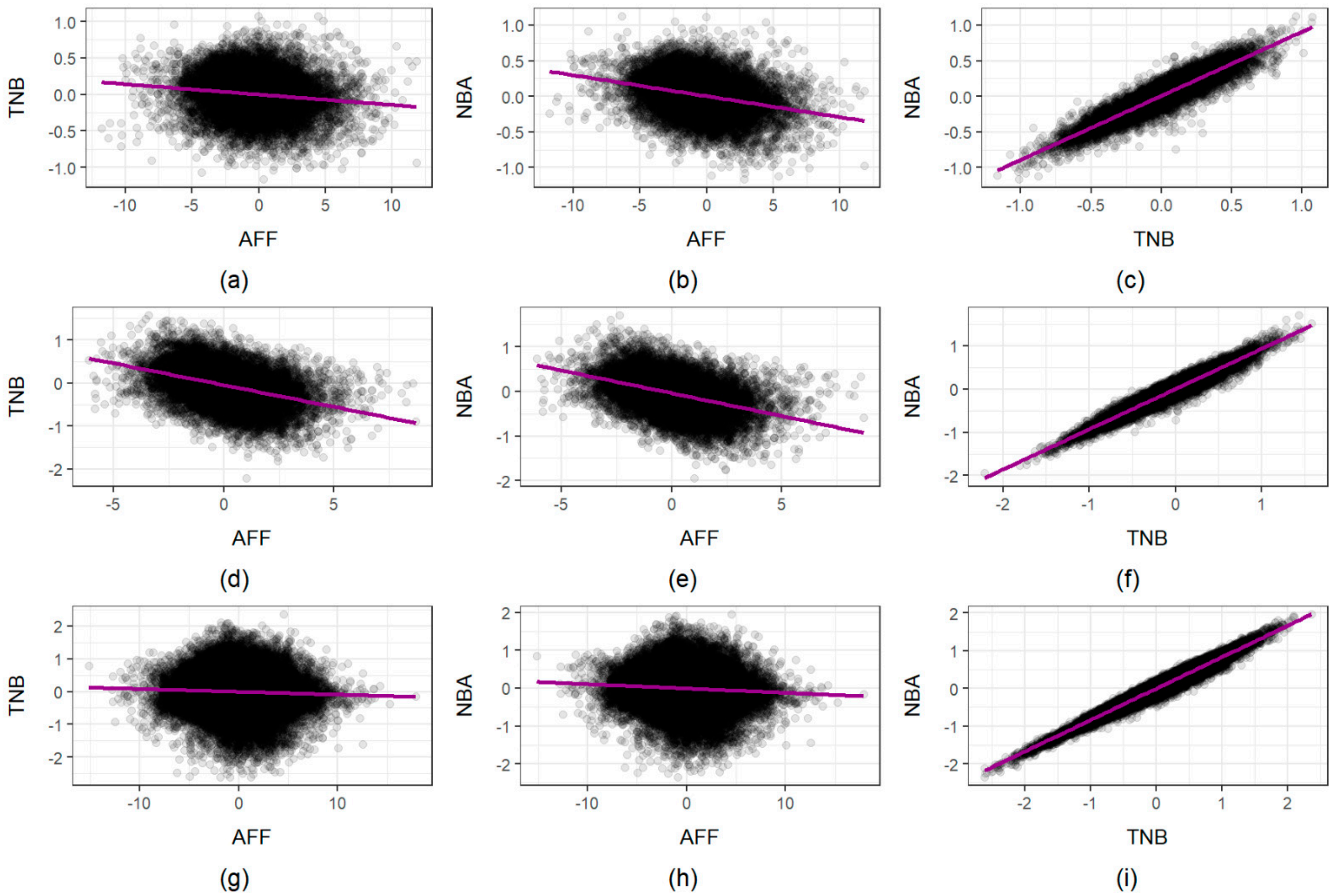

Figure S2. Distribution of EBV for AFF (age at first farrowing), TNB (total number of piglets born), and NBA (total number of piglets born alive) in three Korean pig breeds. (**a**–**c**) Duroc pigs, (**d**–**f**) Landrace pigs, (**g**–**i**) Yorkshire pigs.
